# Conservation genetics of the pond bat (*Myotis dasycneme*) with special focus on the populations in northwestern Germany and in Jutland, Denmark

**DOI:** 10.1002/ece3.5119

**Published:** 2019-04-01

**Authors:** Liselotte Wesley Andersen, Ronja Dirksen, Elena A. Nikulina, Hans J. Baagøe, Gunars Petersons, Péter Estók, Oleg L. Orlov, Maria V. Orlova, Florian Gloza‐Rausch, Matthias Göttsche, Esben Terp Fjederholt, Frauke Krüger, Morten Elmeros

**Affiliations:** ^1^ Department of Bioscience Kalø, Aarhus University Grenå Denmark; ^2^ Population Genetics Group, Department of Biology, Zoological Institute Christian‐Albrechts University of Kiel Kiel Germany; ^3^ Centre for Baltic and Scandinavian Archaeology (ZBSA) Schleswig‐Holstein State Museums Foundation Schleswig Germany; ^4^ Natural History Museum of Denmark Copenhagen Denmark; ^5^ Faculty of Veterinary Medicine Latvia University of Life Sciences and Technologies Jelgava Latvia; ^6^ Eszterházy Károly University Eger Hungary; ^7^ International Complex Research Laboratory for Study of Climate Change, Land Use and Biodiversity University of Tyumen Tyumen Russia; ^8^ Department of Biochemistry Ural State Medical University Ekaterinburg Russia; ^9^ Laboratory of Biodiversity Monitoring National Research Tomsk State University Tomsk Russia; ^10^ Noctalis Fledermaus‐Zentrum GmbH Bad Segeberg Germany; ^11^ Faunistisch‐ Ökologische Arbeitsgemeinschaft, AG Wirbeltiere Christian‐Albrechts‐Universität Kiel Kiel Germany; ^12^ Myotis v. Esben T. Fjederholt Vester Skerninge Denmark; ^13^ Hamann & Schulte Umweltplanung Gelsenkirchen Germany

**Keywords:** conservation genetics, cross‐border management, migration, *Myotis dasycneme*, phylogeny, population structure

## Abstract

Conservation genetics is important in the management of endangered species, helping to understand their connectivity and long‐term viability, thus identifying populations of importance for conservation. The pond bat (*Myotis dasycneme*) is a rare species classified as “Near Threatened” with a wide but patchy Palearctic distribution. A total of 277 samples representing populations in Denmark, Germany, Latvia, Hungary, and Russia were used in the genetic analyses; 224 samples representing Denmark, Germany, and Russia were analyzed at 10 microsatellite loci; 241 samples representing all areas were analyzed using mitochondrial D‐loop and cytochrome B sequences. A Bayesian clustering approach revealed two poorly resolved clusters, one representing the Danish and German groups and the other the Russian group. However, significantly different pairwise *F*_ST_ and *D*_EST_ estimates were observed between the Danish and German groups and between the Danish and Russian groups suggesting a recent population structure. These conflicting results might be attributed to the effect of migration or low resolution due to the number of microsatellite markers used. After concatenating the two mitochondrial sequences, analysis detected significant genetic differentiation between all populations, probably due to genetic drift combined with a founder event. The phylogenetic tree suggested a closer relationship between the Russian and Northern European populations compared to the Hungarian population, implying that the latter belongs to an older ancestral population. This was supported by the observed haplotype network and higher nucleotide diversity in this population. The genetic structuring observed in the Danish/German pond bat stresses the need for a cross‐border management between the two countries. Further, the pronounced mtDNA structuring, together with the indicated migration between nearby populations suggest philopatric female behavior but male migration, emphasizes the importance of protecting suitable habitat mosaics to maintain a continuum of patches with dense pond bat populations across the species' distribution range.

## 1 INTRODUCTION

Conservation genetics is an important tool in the management of endangered species, resolving population connectivity and thereby informing us of the long‐term viability of a species, thus identifying populations in special need of conservation (Pérez‐Espona & ConGRESS Consortium, 2017; Stockwell, Hendry, & Kinnison, 2003).

The pond bat, *Myotis dasycneme *(Boie, 1825), is widely distributed in the temperate lowlands of the Palearctic across northern and central Europe from northern France, Belgium, and the Netherlands toward the east into Asia to the Yenisei region in central Russia (Görföl et al., 2018; Limpens, Lina, & Hutson, 2000; Piraccini, 2016; Strelkov, 1969). Its foraging behavior, feeding on insects associated with water, renders the species dependent on larger, relatively calm bodies of water (Ahlén, Baagøe, & Bach, 2009; Baagøe, 1987; Ciechanowski, Zapart, Kokurewicz, Rusiński, & Lazarus, 2017; Dietz, Helversen, & Nill, 2009; Haarsma & Siepel, 2014; Krüger et al., 2014; Limpens et al., 2000). Consequently, the pond bat has a patchy distribution throughout its range with relatively high densities in regions with suitable habitats, that is, in the Netherlands, northwestern Germany, and in Denmark.

The global status of pond bat is assessed as “Near Threatened” (IUCN, 2018) as its population decline is suspected to approach 30% over the last 15 years (three generations) (Piraccini, 2016). Degradation and loss of aquatic habitats, commuting routes and roosting sites, human disturbance of hibernacula, and water pollution are listed as the main threats for the species (Limpens et al., 2000; Piraccini, 2016).

In Denmark, the pond bat is relatively common in the central and northern parts of Jutland and has a patchy but stable occurrence in the southeastern parts of the country (Baagøe, 2007). Further, there is an increasing number of new records also from other parts of Denmark (H. J. Baagøe, Pers. Comm.). Almost the entire Jutland population (around 8,000 individuals) seems to hibernate in a few old limestone mines, Mønsted and Daugbjerg, from where they disperse to maternity roosts during summer (Baagøe & Degn, 2009). The conservation status of the species is assessed as favorable but the population is vulnerable as it is mainly restricted to a very low number of hibernacula during winter (Baagøe, 2010; www.eionet.eu). In Germany, the pond bat has a fragmented distribution, occurring mainly in the northern and western states (www.bfn.de). Schleswig‐Holstein harbors the biggest known population and a large hibernaculum with more than 1,000 individuals (Jagd & Artenschutz, 2010). The species' conservation status is assessed as moderately unfavorable due to low habitat quality and its restricted population size in some German states (www.eionet.eu; www.eurobats.org). (For species assessment in Latvia, Hungary, and Russia, see Supporting Information Appendix S1).

Studies have investigated the phylogenetic position of the pond bat (Kruskop, Borisenko, Ivanova, Lim, & Eger, 2012; Mayer & von Helversen, 2001; Ruedi & Mayer, 2001), but nongenetic study has addressed the relationship among populations in different parts of its range. Information on population structure/connectivity, as well as genetic diversity, is crucial to understand population processes and fundamental for adaptive processes and species resilience, which is especially important in light of the climate change, impairing pond bat habitat (Meinig, 2010).

Pond bats may migrate more than 300 km between summer and winter habitats (Hutterer, Ivanova, Meyer‐Cords, & Rodrigues, 2005), but bats generally show a high site fidelity to roost sites (Altringham, 2011). Ringing efforts to track migration were conducted in Jutland limestone mines, Denmark, in the 1950s and 1960s (Egsbæk & Jensen, 1963) and in Germany in 2008 (F. Gloza‐Rausch, Pers. Comm.). In the first study, no recapture was recorded in northern Germany, and further, to the authors' knowledge, bats ringed in northern Germany have not been recorded from the four limestone mines in Jutland (H. J. Baagøe, Pers. Comm.). In 2008, a female pond bat ringed as a subadult in Methorst, northern Germany, was resighted hibernating in Mønsted limestone mines spring 2009 in Denmark, a migration distance of 250 km (F. Gloza‐Rausch, Pers. Comm.). In January 2010, this bat hibernated in a bunker in Schafstedt, northern Germany, 250 km south of Mønsted, and the following summer, it bred in its natal roost site in Methorst (Jagd & Artenschutz, 2010). This observation may indicate that there is some dispersal and cohesion among the pond bat populations of Jutland and Schleswig‐Holstein.

The objective of the present study was to investigate the genetic population structure of pond bat mainly in Denmark and Germany (including fewer samples from Latvia, Hungary, and Russia) based on variation in ten microsatellite markers, the control region (CR), and cytochrome B (CytB) of the mitochondrial DNA (mtDNA). The microsatellite markers were used to (a) assess the genetic diversity, (b) estimate population structure, and (c) evaluate immigration/emigration rate and direction (gene flow) within the populations in Denmark and Germany and between them and samples from Russia using assignment test and exploring migration network based on various population structure estimates. Further, as pond bat populations have declined during the last decades (Piraccini, 2016), we tested if this was reflected in the species' genetic makeup. mtDNA markers were used to quantify genetic diversity and population structure among all the sampled populations, but also to try to uncover demographic history, for example, in terms of former population expansions despite small, unequal sample sizes.

## 2 MATERIALS AND METHODS

### 2.1 Sampling

**Figure 1:**
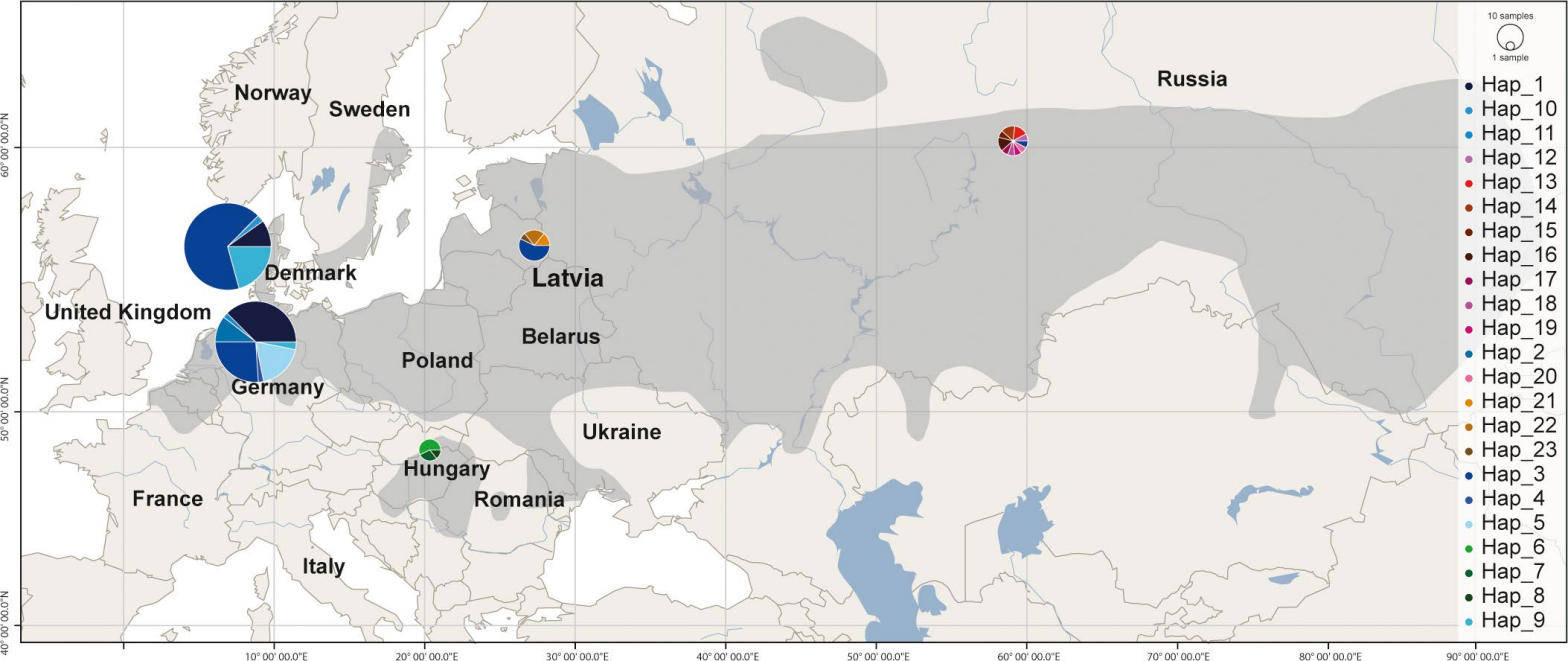
Map showing the global distribution (gray shaded area) of the pond bat together with sampling localities and the haplotype diversity for the concatenated CytB‐CR region in mtDNA

In Germany, samples were collected from bats caught using mist nets placed in foraging areas or close to nursery roosts and at major hibernacula in northern Germany (Table 1). In Denmark, samples were collected from bats caught with harp traps during emergence from the large hibernacula in the limestone mines in Mønsted and Daugbjerg. In Hungary, samples were collected near a lake and at three swarming sites situated in the Bükk Mountains in the northern part of the country. In Russia, samples were collected at two major hibernacula in the Smolinskaya and Arakaevskaya caves in the Middle Urals, while the samples from Latvia were fecal samples collected from the ground (Table 1; Figure 1). The samples from Latvia and Hungary were not genotyped at the microsatellite markers due to poor DNA quality (Latvian samples) and low sample size (Hungarian samples). Given the substantial temporal difference between sampling years for the Danish localities suggesting additional impact of genetic drift on the analysis, these samples were kept separate during the initial analysis.

**Table 1:** Sampling area and year, colony sites and type, and number of samples and types analyzed for microsatellites, control region (CR), and cytochrome B (CytB) in mtDNA, DNA source for the pond bat

Country	Colony site	Colony type	Microsatellite	Control region	CytB	Sample type	Sampling year
Denmark	Mønsted^a^	Hibernaculum	51	48	50	Wing	2003
	Mønsted^b^	Hibernaculum	19	20	18	Saliva	2011
	Daugbjerg^c^	Hibernaculum	38	34	34	Wing	2009
	Daugbjerg^d^	Hibernaculum	12	12	12	Saliva	2011
Germany	Wahlstorf	Maternity roost	28	29	30	Wing	2009
	Methorst	Maternity roost	21	21	21	Wing	2009
	Groß Nordsee	Maternity roost		7	7	Wing	2009
	Ratekau	Maternity roost		8	8	Wing	2010
	Bad Segeberg	Hibernaculum	32	38	40	22 Wing 10 Saliva 8 Feces	2011
Latvia	Diverse	Maternity roost	Not used	25	19	Feces	2011
Hungary	Diverse	Hibernaculum	Not used	8	7	Wing	2011
Russia	Diverse	Hibernaculum	23	24	14	Wing	2010 and 2011

a MON03.

b MON11.

c DAU09.

d DAU11.

From some of the pond bats (see Table 1), samples were taken by puncturing the wing membrane of the chiropatagium with a stanza (3 mm diameter; Worthington & Barratt, 1996). These samples were stored in absolute ethanol (99%) at 4°C or a saturated salt/DMSO solution. Saliva samples were taken with Isohelix DNA buccal swabs (Cell Projects, Kent, UK; see Table 1). The swabs were stored in the provided tubes (Isohelix, Cell Projects, Kent, UK) and frozen immediately at −20°C.

### 2.2 Laboratory procedures

DNA from wing samples was extracted using Qiagen DNeasy^®^ Tissue Kit following the manufacturer's protocol (QIAGEN). DNA from buccal samples was extracted using the Isohelix DNA Isolation Kit following the manufacturer's protocol (Isohelix, Cell Projects Ltd). From fecal samples, DNA was extracted using the Biolab Products Crystal Stool DNA Kit (Biolab Products) adjusting the manufacturer's protocol for a smaller amount of sample. The extraction was conducted in the ancient‐DNA laboratory at the Centre for Baltic and Scandinavian Archaeology (ZBSA), Schleswig‐Holstein State Museums Foundation, Germany.

Ten microsatellite markers developed for *Myotis myotis* (Castella & Ruedi, 2000) (Supporting Information Appendix S2) were PCR‐multiplexed in two runs using the QIAGEN Multiplex PCR kit in a 12.5 µl reaction volume with an annealing temperature of 57°C and conditions following the manufacturer's protocol (QIAGEN). Mix 1 consisted of A13, E24, F19, G9, G25, and H29 and Mix 2 of D9 and H19. D15 and G30 were run separately. The PCR products were analyzed using an ABI PRISM 3730 DNA sequencer and genotyped in GeneMapper^®^ version 4.2 (Applied Biosystem).

A 521 bp part of the mitochondrial cytochrome b and 247 bp of the D‐loop region of mitochondrial control region were amplified using the following primers: MyoF ATGACCAACATTCGAAAATCTC, MyoR ATGTTAAAGTTAGGAGATCTGC; and MyCR‐F TTAATTACTAATCAGCCCATGCC, MyCR‐R1 GTTGTTGTGTTGTATGTCCTG. Amplification was conducted in a reaction volume of 12.5 µl using Amplicon DNA polymerase master mix (AMPLICON) and 10 µM of each primer, in a standard PCR using one cycle for 5 min at 95°C, 35 cycles at annealing temperatures of 48 and 41°C, respectively, and extension time for 7 min at 72°C. DNA extractions were stored at −20°C. The amplified DNA strands obtained from German, Latvian, Hungarian, and Russian samples were Sanger‐sequenced one way at the Institute of Clinical Molecular Biology, Kiel University, Germany, while the amplified DNA strands from the Danish samples were sequenced forward and reverse at MACROGEN Inc. (Amsterdam, Holland). The sequences were later analyzed using Sequencher 5.3 (Gene Code).

### 2.3 Data analysis

#### 2.3.1 Genetic variation

Genetic variation, estimated as observed and expected heterozygosity, and tests for goodness of fit to the Hardy–Weinberg equilibrium were performed in FSTAT (Goudet, 1995) and GenAlEx 6.5 (Peakall & Smouse, 2006). The presence of null alleles in the microsatellite loci was checked using MICRO‐CHECKER 2.2.1 (Van Oosterhout, Hutchinson, Wills, & Shipley, 2004) for all except Daugbjerg 2009. Genotypic linkage disequilibrium was tested between all pairs of the 10 loci using GENEPOP version 3.4 with 5,000 iterations (Raymond & Rousset, 1995). Genetic variation in the CytB and D‐loop (CR) sequences and the concatenated CytB‐CR sequences was estimated as haplotype diversity (HD) and nucleotide diversity (π) using DnaSP v5 (Librado & Rozas, 2009).

#### 2.3.2 Population structure

The number of groups represented in the three locations was estimated in STRUCTURE version 2.3.4 (Pritchard, Stephens, & Donnelly, 2000) using data from only seven microsatellite markers due to state (low genetic variation and linkages disequilibrium; see “Results”). This software uses a Bayesian approach by clustering individuals, minimizing Hardy–Weinberg disequilibrium and gametic phase disequilibrium between loci. The analysis was conducted using the admixture model and the model of correlated allele frequencies between clusters. Following the recommendation by Wang (2017), alpha was adjusted to 0.33 according to the number of clusters, *K*, expected (*K* = 3 in this instance) to account for the unbalanced sample size. The results of the tests were based on 1,000,000 iterations, a 100,000 burn‐in period. All samples were combined, and STRUCTURE was run with *K* = 1 to *K* = 7 and 10 replicates without prior information regarding the sample's origin. The clusters of individuals forming the number of populations with the highest likelihood were assigned to sampling locations. As it might be difficult to infer the number of clusters represented due to an effect of isolation by distance (IBD) and extensive admixture (Falush, Stephens, & Pritchard, 2003; Pritchard et al., 2000), KFinder (Wang, 2019) was applied to infer the number of clusters. KFinder includes three different criterions for assessing the most likely number of populations represented by the sample of individuals. The first two are the classical methods, Pr[X|K] method (Pritchard et al., 2000) and the Δ*K* method (Evanno, Regnaut, & Goudet, 2005), and the third is the Parsimony Index, which chooses the *K *that repetitively returns the minimal mean admixture of individuals (Wang, 2019). Wang (2019) shows that this method often performed better in returning the correct population structure compared to the two former methods. Further, CLUMPAK software was applied to visualize the STRUCTURE results over the number of runs (Kopelman, Mayzel, Jakobsson, Rosenberg, & Mayrose, 2015).

Pairwise multilocus *F*_ST_ (Weir & Cockerham, 1984) and *D*_EST_ (Jost, 2008) were calculated using the R package diveRsity (Keenan, McGinnity, Cross, Crozier, & Prodöhl, 2013) and statistically evaluated after 1,000 bootstraps. The values obtained were regarded as significant when the confidence interval around the estimate did not contain zero. Both *F*_ST_ and *D*_EST_ vary between 0 and 1 (no differentiation–complete differentiation). *F*_ST_ is dependent on the variability of the used markers, while *D*_EST_ is independent of this within‐population diversity (Jost, 2008; Verity & Nichols, 2014).

Discriminant analysis of principal components (DAPC; Jombart, Devillard, & Balloux, 2010) was used to further explore the possibility of group structure in the data not recovered by STRUCTURE. This method uses the genetic relationships among individuals to identify groups and is based on allele frequencies of the microsatellite markers, and does not rely on model assumptions. This was conducted using the adegenet package (Jombart, 2008) in R (www.r-project.org; R Development Core Team, 2008).

Population structure based on variation in the mtDNA sequences was examined using the pairwise distance between haplotypes as genetic distance and Φ_ST_ statistics. As both CytB and CR are situated in the mitochondria, the two sequences were concatenated for the single individuals to represent a 768‐bp sequence. Mitochondria represents one marker, and it has been shown (Jacobsen et al., 2012) that the longer sequences give higher resolutions. The tests were run for 10,000 permutations over individual haplotypes among potential populations/subpopulations in ARLEQUIN 3.5.1 (Excoffier & Lischer, 2010). The sequential Bonferroni procedure was applied using a significance level of 5% whenever multiple tests were performed (Rice, 1989).

To test if a possible population structure pattern could be explained by pure IBD, genetic distance in terms of Φ_ST_ for the concatenated mitochondrial sequences and geographical distance between the areas were computed in IBD (Bohonak, 2002). Genetic distance based on microsatellite markers was not included due to the low number of populations analyzed with them. Geographical distances were measured in kilometers drawing straight lines between the sampling localities using Google™ Earth.

#### 2.3.3 Migration and detection of first‐generation migrants

An assignment test was conducted to allocate individuals to the population from which they most likely originate. This was performed in GENECLASS2 (Piry et al., 2004) that uses the individual's multilocus genotype likelihoods to identify population origin (Paetkau, Slade, Burden, & Estoup, 2004). Further, to detect first‐generation migrants (FGM) in the bat populations, the likelihood computation, L = L_home/L_max_not_home, was used (Paetkau et al., 2004; Piry et al., 2004). For both tests, levels of significance were determined comparing the assigned individuals' genotypes with a simulated set (10,000) obtained using the allele frequencies from the different areas (Paetkau et al., 2004). The exclusion probability (at the 5% level) of a population as the origin and the probability that an individual is a migrant were calculated based on the resampling algorithm of Paetkau et al. (2004). The assignment test was applied including only German and Danish samples to avoid bias due to differences in sampling size (Paetkau et al., 2004).

To analyze migration direction, DivMigrate implemented in the diveRsity R package (Keenan et al., 2013; Sundqvist, Keenan, Zackrisson, Prodöhl, & Kleinhans, 2016) was applied. The method uses the geometric means of allele frequencies and the genetic differentiation between pairs of populations to deduced migration rate and direction. The relative migration network is illustrated as a graph showing the gene flow between the populations. The relative migration network was estimated using the pairwise population differentiation estimates in terms of D_EST_ (Nei, 1973; Nei & Chesser, 1983; Sundqvist et al., 2016). Significant asymmetrical migration was tested based on 1,000 bootstraps in DivMigrate (Keenan et al., 2013; Sundqvist et al., 2016).

### 2.4 Population demography

#### 2.4.1 Bottleneck

##### Microsatellite

During a bottleneck, population size declines abruptly, which is expected to affect the number of alleles faster than loss of heterozygosity. This will cause heterozygote excess in the population (Cornuet & Luikart, 1996). The microsatellite dataset from the Danish, German, and Russian pond bat groups was used in the bottleneck test performed in BOTTLENECK 1.2 (Piry, Luikart, & Cornuet, 1999). The nonparametric Wilcoxon's test was used to evaluate if the number of loci with heterozygote excess is larger than expected to occur by chance. The tests were performed assuming the two‐phase mutation model (TPM) (Di Rienzo et al., 1994), with single‐step mutations ranging from 70, 90, 95, and 99% and a 12% variance of multistep mutations as recommended (Piry et al., 1999).

#### 2.4.2 Demographic inferences

##### mtDNA

Historical population fluctuations were explored using the concatenated CytB‐CR sequences for all samples. This was performed using tests of population growth and mismatch analysis (Schneider & Excoffier, 1999) using Tajima's D test of selective neutrality (Tajima, 1989), Fu's *F*_s_ (Fu, 1997), and distribution of pairwise differences of nucleotide sequences (mismatch distribution) (Rogers & Harpending, 1992). The detection of excess numbers of singleton mutations relative to expectations under the standard neutral model is indicative of recent population growth. This will be uncovered as significantly negative values of both estimates. The Raggedness Index (Harpending, Sherry, & Rogers, 1993) reflects the mismatch distribution of pairwise nucleotide differences between haplotypes. If the distribution pattern is multimodal or “ragged,” the population is expected to be stable or declining slowly (Rogers & Harpending, 1992; Slatkin & Hudson, 1991). The mismatch distributions were tested by estimating the goodness of fit between observed and expected distributions using the parametric bootstrap (1,000) approach in ARLEQUIN 3.5.1 (Excoffier & Lischer, 2010). The sum of square deviations (SSDs) was the test statistic used between observed and expected distributions, where *p*‐values were calculated as the proportion of simulations producing a larger SSD than the observed SSD.

#### 2.4.3 Phylogeny

The relationships among the observed mtDNA haplotypes for CytB and CR as well as the concatenated sequence were estimated based on a median‐joining network, which allows intermediate haplotypes in the network (Bandelt, Forster, & Rohl, 1999). The network was generated using DnaSP (Librado & Rozas, 2009) and POPART (Leigh & Bryant, 2015). Further, a phylogenetic consensus tree was inferred using the Bayesian method implemented in MrBayes v3.2.6 (Ronquist et al., 2012) and the HKY model that was found to best fit the concatenated dataset (jModelTest; Posada, 2008) (data not shown). The concatenated sequence of CytB and CR (GenBank accession number KT901455 whole mitogenome) from the greater mouse‐eared bat, *Myotis myotis*, was included as an outgroup.

MrBayes was run twice using the default settings involving two independent MCMC runs in each and four chains. The default sample frequency of 500 and diagnostic frequency of 5,000 and run length of 1,000,000 were used, discarding 25% of the samples of burn‐ins and run until the standard deviation of split frequencies was below 0.01, which is indicative of convergence according to the authors (Ronquist et al., 2012). The obtained topology and branch lengths of the tree were visualized in FigTree (http://beast.bio.ed.ac.uk/software/FigTree).

## 3 RESULTS

### 3.1 Genetic diversity

Species identification of fecal samples was based on alignment of CR and CytB sequences to the known sequences obtained from the tissue samples from live specimens in the study, and all were confirmed to be pond bats (data not shown).

The datasets used to estimate genetic diversity, population structure, and migration based on the 10 microsatellite markers (Supporting Information Appendix S2) and the two mtDNA markers, CR (247 bp) and CytB (521 bp) sequences, and the concatenated sequences (768 bp) for the different localities are given in Table 1.

For the microsatellites, significant departures from Hardy–Weinberg expectations in terms of heterozygote deficiency were observed at locus *D9* and *D15* in the Danish sample (Supporting Information Appendix S3). The observed microsatellite genetic diversity was at the same level for the Danish (*H*_E_ = 0.676 ± 0.092), German (*H*_E_ = 0.673 ± 0.094), and Russian samples (*H*_E_ = 0.659 ± 0.095). Analysis for genotypic linkage disequilibrium revealed that one pair of loci (*G9* and *H29*) showed linkage (all populations) (data not shown). At the mtDNA level, the highest level of genetic diversity in the CR was observed in the Russian sample (HD = 0.841 ± 0.01, π = 0.0057), while the lowest level for both estimates was observed in the Latvian sample where only one haplotype was found. Unique mt haplotypes were observed in all populations except Latvia; Russia had seven while Denmark and Germany had one and two unique haplotypes, respectively, and Hungary three (Supporting Information Appendix S4). A common haplotype Pb_Gecr3 (Supporting Information Appendix S5a) was found in all sampling areas except Hungary. A slightly different diversity pattern was observed in the CytB sequences, where the Russian sample still contained the highest diversity (HD = 0.868 ± 0.014, π = 0.0029) and five unique haplotypes, but only two haplotypes and low nucleotide diversity (HD = 0.335 ± 0.004, π = 0.0006) were found in the Danish sample despite the large sample size. Pb_Gec1, the most common haplotype, was observed in all areas except Hungary (Supporting Information Appendix S5b).

For the concatenated mtDNA sequence the Russian sample had the highest genetic diversity (HD = 0.962 ± 0.011, π = 0.004), then the German population (HD = 0.752 ± 0.002, π = 0.0023), except for the nucleotide diversity where the Hungarian sample had the second highest (HD = 0.667 ± 0.06, π = 0.0026). The Danish sample had the lowest diversity (HD = 0.507 ± 0.004, π = 0.0007) (Table 2).

**Table 2:** Sample size (*N*), genetic diversity (*H*_O_, *H*_E_; GenAlEx; Peakall & Smouse, 2006,2012), standard error (*SE*), and *F*_IS_ (deviation from Hardy–Weinberg expectations) based on 10 microsatellites (FSTAT; Goudet, 1995)

	MON03	MON11	DAU09	DAU11	Denmark Total	Germany	Latvia	Hungary	Russia
Microsatellites									
*N*	51	19	38	12	120	81	0	0	23
*H*_O_	0.647	0.608	0.647	0.598	0.636	0.651	0	0	0.647
*SE*	0.095	0.089	0.093	0.108	0.090	0.096	0	0	0.097
*H*_E_	0.652	0.659	0.665	0.643	0.676	0.673	0	0	0.659
*SE*	0.095	0.084	0.090	0.084	0.092	0.094	0	0	0.095
*F*_is_	0.018	0.106	0.039	0.114	0.063	0.038	0	0	0.041
CytB‐CR concatenated									
*N*	49	18	32	12	111	96	14	7	13
H	4	2	4	3	4	7	4	3	10
S	3	1	3	2	3	11	3	4	11
Singleton	1	0	0	0	0	0	1	1	4
P shared	2	1	3	2	3	11	2	3	7
HD	0.504	0.425	0.573	0.545	0.507	0.752	0.648	0.667	0.962
*SE*	0.07	0.100	0.084	0.144	0.004	0.002	0.031	0.06	0.011
$\Phi_{\text{wa}}\left(\%\right)$	0.08	0.040	0.1	0.09	0.07	0.28	0.12	0.21	0.46
π (%)	0.07	0.060	0.09	0.08	0.07	0.23	0.1	0.26	0.4
Tajima D	−0.35	0.87	−0.26	−0.248	−0.01	−0.465	−0.565	1.076	−0.504
Fu's *Fs*	−0.584	1.039	−0.52	−0.269	−0.038	0.716	−0.99	1.321	**−4.98**
Spatial expansion									
SSD	**0.015**	**0.01**	**0.019**	**0.022**	**0.014**	0.035	**0.032**	0.073	0.007
Ragg. Id.	0.16	0.203	0.167	0.185	0.158	0.081	0.211	0.283	0.043
Demographic expansion									
SSD	**0.016**	**0.009**	0.018	0.022	**0.015**	**0.027**	0.03	0.111	0.007
Ragg. Id.	**0.16**	0.203	0.167	0.185	**0.158**	0.081	0.211	0.283	0.043

Note

H = genetic diversity as the number of mtDNA haplotypes; HD = haplotype diversity; *N* = sample size; π = nucleotide diversity (Nei, 1987); S = number of segregating sites; Singleton = mutation observed in only one sequence; P shared = mutation observed in at least two sequences (DnaSP; Librado & Rozas, 2009). Tests for selective neutrality, Tajima's D (Tajima, 1989) and Fu's *F_s_* (Fu, 1997) (in ARLEQUIN; Excoffier & Lischer, 2010), for the five different pond bat regions were performed. The Danish samples were divided according to locality and year where MON03 = Mønsted 2003, MON11 = Mønsted 2011, DAU09 = Daugbjerg 2009, and DAU11 = Daugbjerg 2011. Population expansion indices estimated for the concatenated sequences in terms of SSD (sum of squares deviations) between observed and expected mismatch and Ragg. Id. (Raggedness Index) of the mismatch distribution (ARLEQUIN; Excoffier & Lischer, 2010). Bold = significant at the 5% level. For *F*_s_, *p* = 0.018.

### 3.2 Population structure

#### 3.2.1 Microsatellites

**Figure 2:**
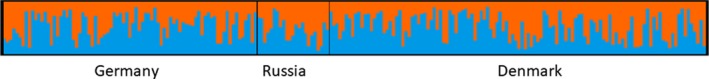
Graphical output from STRUCTURE (Pritchard et al., 2000) after evaluation of the number of clusters present in the samples using KFinder (Wang, 2019) and processed in CLUMPAK (Kopelman et al., 2015) illustrating the population structure (STRUCTURE; Pritchard et al., 2000) based on seven microsatellite markers for the three pond bat localities, Denmark, Germany, and Russia without using prior knowledge of locality. Each vertical line represents an individual, and the color composition displays the probability of belonging to a clusters

Locus *G9 *was omitted due to the observed significant genotypic linkage to *H29*, and *G25* and *H19* were omitted due to occurrence of observed rare alleles and low variability. It has been shown by Linck and Battey (2019) that occurrences of rare alleles introduce noise when estimating population structure, blurring the population inference. Consequently, STRUCTURE analysis together with all the data analysis based on microsatellite markers were performed based on the remaining seven loci and the aggregated Danish samples. The three different approaches implemented in KFinder (Wang, 2019) to identify the individuals' ancestry from the STRUCTURE (Pritchard et al., 2000) results, all returned two populations (best *K* = 2) as the most probable structure (Table 3; Figure 2). The structure found was not clear, but inspecting the output files suggested that Denmark and Germany belonged to one population and Russia to another population (data not shown).

**Table 3:** Estimation of the most likely number of populations present in the sample of pond bats based on the Bayesian method implemented in STRUCTURE

*K*	STRUCTURE	STRUCTURE HARVESTER	KFinder	
	Mean Pr[*X*|*K*]	Δ*K*	Parsimony Index	Best *K*
1	−6,044.16	—	0.5	
**2**	**−6,027.46**	**12.8699**	**0.6822**	**2**
3	−6,057.61	3.0464	0.4771	
4	−6,113.61	9.3609	0.4262	
5	−6,454.46	0.4418	0.3529	
6	−6,771.41	2.1273	0.0967	
7	−6,862.61	—	0.2878	

Note

Three different approaches were applied to interpret the best number of clusters (best *K*) obtained from STRUCTURE: the mean likelihood Pr[X|K] that maximize *K* (Pritchard et al., 2000), *ΔK* (the largest rate of change of the log probability given the data) (Evanno et al., 2005; STRUCTURE HARVESTER (Earl & VonHoldt, 2012)), and the Parsimony Index (KFinder, Wang, 2019), identifying the *K*, which repeatedly returns the minimal mean admixture of the sample. *K* = number of assumed clusters. The analysis was run for *K* = 1 to *K* = 7 and 10 replications. All estimated in KFinder (Wang, 2019).

**Figure 3:**
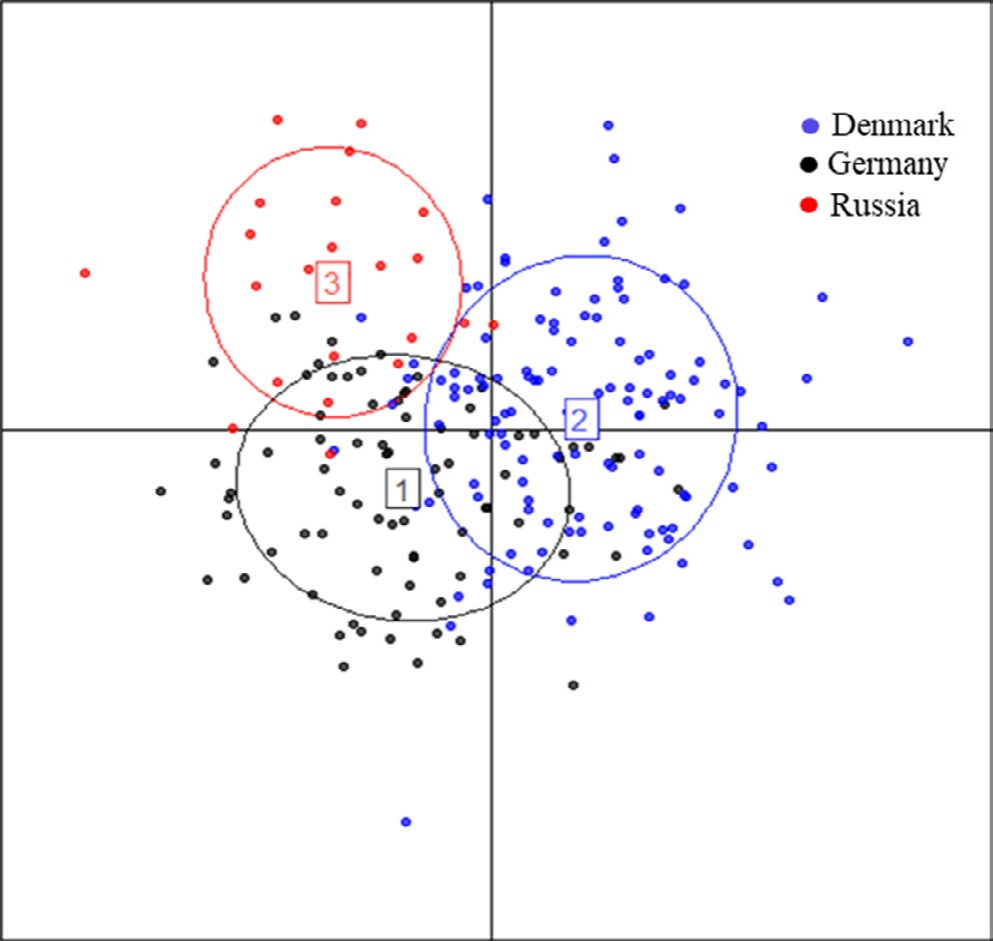
Discriminant analysis of principal components (DAPC; Jombart et al., 2010) based on seven microsatellite markers identifying three genetic clusters of pond bats from Germany (cluster 1), Denmark (cluster 2), and Russia (cluster 3)

Population structure based on pairwise *F*_ST_ and *D*_EST_ estimates revealed significant genetic differentiation between the Danish and German pond bats (Table 4a). A temporal effect was observed in the Danish sample, which was most pronounced for the *F*_ST_ method (Table 4b) but not observed in the STRUCTURE analysis. Using DAPC (Figure 3), three groups were identified. The first and second DF (discriminant factor) discriminated between Russian and Danish–German areas, while only the first DF discriminated between the Danish and German pond bats (and to a lesser degree and with some overlap).

**Table 4:** Genetic divergence estimated between the populations based on seven microsatellite markers and concatenated mtDNA sequences and the different combinations of geographical regions of the pond bat

(a) Microsatellites, DK samples pooled						
	Denmark	Germany	Russia			
Denmark		**0.018**	**0.057**			
Germany	***0.004***		0.033			
Russia	**0.011**	0.005				

(b) Microsatellites, DK samples divided according to year and location						
	MON03	MON11	DAU09	DAU11	Germany	Russia
MON03		0.008	***0.038***	0.011	0.023	**0.07**
MON11	***0.014***		0.055	−0.016	0.021	0.063
DAU09	***0.012***	**0.025**		0.046	***0.035***	**0.085**
DAU11	***0.014***	−0.006	**0.025**		0.024	0.046
Germany	***0.004***	***0.015***	***0.009***	***0.016***		0.033
Russia	***0.013***	**0.025**	***0.012***	**0.024**	0.005	

(c) CytB‐CR concatenated, DK samples divided according to year and location						
	MON03	MON11	DAU09			
MON11	−0.007					
DAU09	−0.043	0.011				
DAU11	−0.022	0.005	−0.05			

(d) CytB‐CR concatenated						
	Denmark	Germany	Latvia	Hungary		
Germany	**0.137**					
Latvia	**0.147**	**0.121**				
Hungary	**0.914**	**0.796**	**0.855**			
Russia	**0.544**	**0.35**	**0.319**	**0.72**		

(e) CytB‐CR concatenated keeping Methorst separate						
	Denmark	Germany	Methorst	Latvia	Hungary	
Germany	**0.184**					
Methorst	**0.412**	**0.26**				
Latvia	**0.147**	**0.151**	**0.368**			
Hungary	**0.914**	**0.797**	**0.862**	**0.855**		
Russia	**0.544**	**0.361**	**0.413**	**0.319**	**0.72**	

(a) Pairwise multilocus F_ST_ below diagonal, *D*_EST_ above diagonal based on seven microsatellites between pond bats from Denmark (diveRsity; Keenan et al., 2013), Germany, and Russia. (b) Multilocus F_ST_ below and *D*_EST_ above diagonal dividing DK samples according to year and location. (c) Pairwise Φ_ST_ results (pairwise distance) based on CytB‐CR concatenated sequences keeping DK sample divided into year and location. (d) Pairwise Φ_ST_ results (pairwise distance) based on CytB‐CR concatenated sequences from the five geographically different regions. (e) Pairwise Φ_ST _results based on CytB‐CR concatenated sequences when separating the German pond bats into two different areas (ARLEQUIN; Excoffier & Lischer, 2010). Bold values for F_ST_ and *D*_EST_ estimates are significant after 1,000 bootstraps (bias‐corrected (Keenan et al., 2013); bold italic values are marginally significant (lower 95% BC_bound approaching 0) after 1,000 bootstraps. Bold values for Φ_ST_ are significant after sequential Bonferroni correction (Rice, 1989).

#### 3.2.2 mtDNA

The population structure analysis in terms of pairwise Φ_ST_ estimates based on the concatenated CytB‐CR sequences did not detect a temporal effect in the Danish samples (Table 4c). Pooling the Danish samples accordingly suggested all analyzed populations were significantly genetically different (Table 4d). This pattern was repeated based on the CytB sequences alone, while the CR region did not separate Denmark and Latvia (Supporting Information Appendix S6a, b). One of the German nursery roosts contained a significantly different haplotype composition compared to the rest of the areas, and to other German pond bats. This different haplotype composition could be attributed to the control region sequences in this sample where haplotype Pb_Gecr5 (Supporting Information Appendix S5a) was more frequently observed.

The results of IBD were nonsignificant despite the method applied, Φ_ST_ or Φ_ST_/(1 − Φ_ST_) (data not shown).

### 3.3 Migration and detection of first‐generation migrants

#### 3.3.1 Microsatellites

The result of the assignment test (Table 5, merging the Danish samples) (Paetkau et al., 2004), showed that ~35% of the pond bat sampled in Germany had the highest probability of belonging to the German population, while ~18% had the highest probability of belonging to the Danish population. For ~39% of the pond bats sampled in Germany, the probability of belonging was inconclusive meaning that the probability was >0.05 and <0.6. Of the pond bats sampled in Germany, ~5% were identified as statistically not belonging to the German population, ~5% could be rejected as coming from the Danish population, and ~6% did not belong to either the German or the Danish population. For Denmark, ~59% of the sampled pond bats had the highest probability of belonging to the Danish population, ~6% to the German population, and ~29% were inconclusive. Among the Danish pond bats, ~17% could be rejected at the 5% level as belonging to the German population, none were rejected as belonging to the Danish population, and ~6% was rejected as belonging from either the Danish or the German population.

**Table 5:** Results of assignment test and detection of first‐generation migrants based on seven microsatellite markers (GENECLASS2; Piry et al., 2004)

	Denmark	Germany	Not GE or DK	Inconclusive
Denmark				
Highest probability of belonging	59.17%	5.83%	NA	29.17%
Significantly rejected at the 5% level	0	17.50%	5.83%	NA
Detection of first‐generation migrants from GE	4/120	NA	NA	NA
Germany				
Highest probability of belonging	18.50%	35.80%	NA	39.51%
Significantly rejected at the 5% level	4.93%	4.93%	6.17%	NA
Detection of first‐generation migrants from DK	NA	7/81	NA	NA

Note

Highest probability of belonging is defined by *p *≥ 0.6. First‐generation migrants at *p *≤ 0.05.

Seven out of the 81 sampled pond bats in the German population were identified to be putative FGM from Denmark, which was more than expected by chance (type 1 error, 5% of 81). Among the Danish samples, four out of 120 sampled pond bats were possible FGM from Germany, but this might be due to chance alone (type 1 error, 5% of 120).

**Figure 4:**
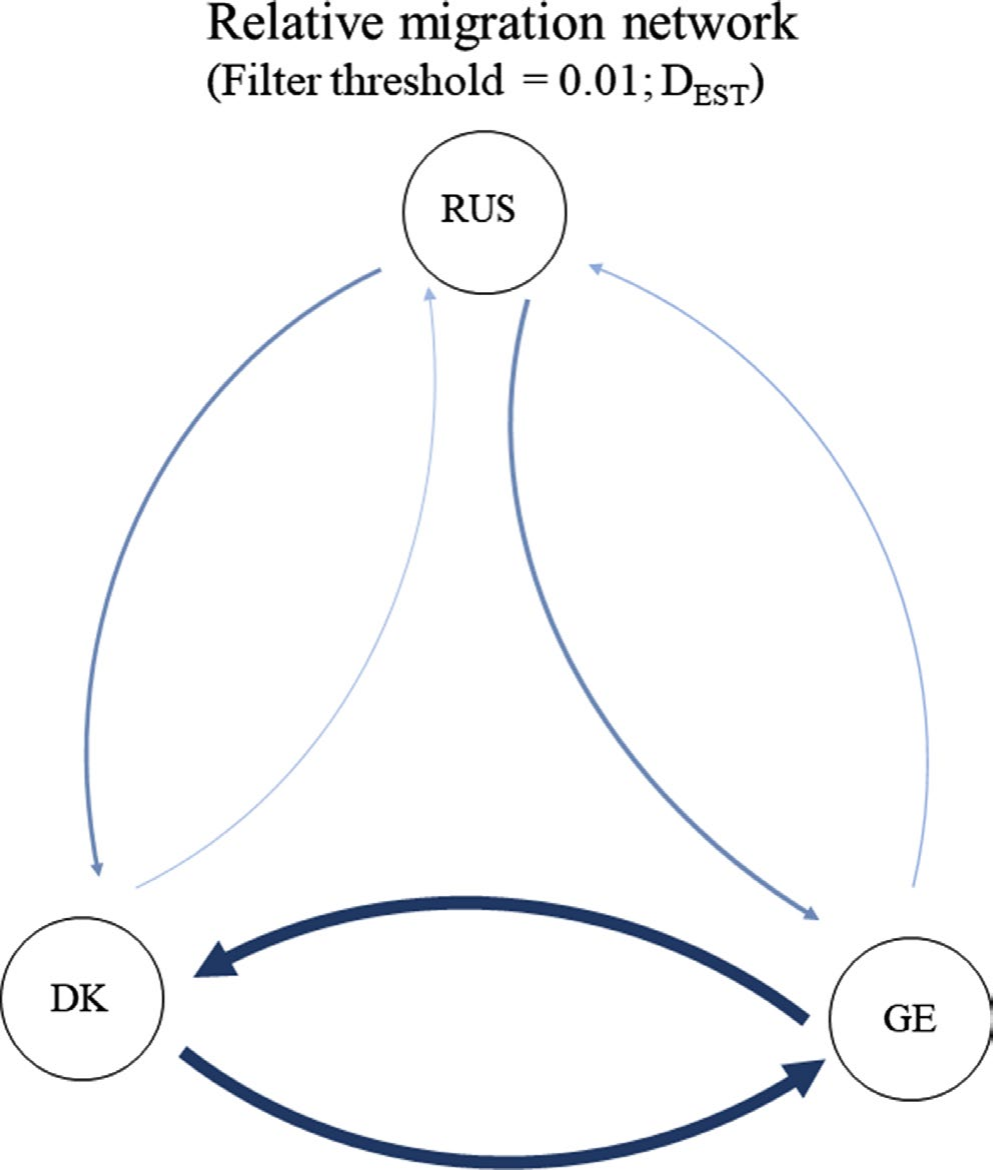
Directional relative migration network illustrating the gene flow connecting the groups of pond bats from Germany (1), Denmark (2), and Russia (3) based on *D*_EST_ estimates. Line thickness and shade between the populations grow with the relative strengths of the gene flow (DivMigrate; Keenan et al., 2013, Sundqvist et al., 2016)

Assessment of the migration direction including the Russian sample using DivMigrate Network (Figure 4) suggested a relative migration network illustrating bidirectional gene flow between Denmark and Germany with a lower gene flow to Russia despite the estimate used. No significant direction of the relative migration was observed between Denmark and Germany.

### 3.4 Population demography

#### 3.4.1 Microsatellite

No bottleneck effects were observed in the Danish and German pond bat populations (Table 6) despite the different percentage of single‐step and multistep mutations applied (data only shown for the pooled DK sample). In the Russian sample, a significant heterozygote excess was observed in some of the tests which might imply a bottleneck effect.

**Table 6:** Bottleneck tests in terms of heterozygote excess evaluated using Wilcoxon's nonparametric signed‐rank test and the mutational model, TPM, with a range of single‐step and multistep mutations (BOTTLENECK; Piry et al., 1999)

	TPM	*p*‐value
Denmark	70	0.2891
Germany		0.1484
Russia		**0.0039**
Denmark	90	0.6563
Germany		0.1484
Russia		**0.0078**
Denmark	95	0.7656
Germany		0.1484
Russia		**0.0195**
Denmark	99	0.9453
Germany		0.1875
Russia		0.0547

Note

*p*‐values significant at the α < 0.05 level are highlighted in bold.

#### 3.4.2 mtDNA

The demographic population history of the investigated areas provided signs of population expansion (Table 2). A significant negative *F*_s_ estimate (Fu, 1997) in the Russian sample indicated a sign of population expansion, which was reflected in the Raggedness Index. This did not reject the null hypothesis of exponential growth (*p* > 0.05). The opposite was observed in the Danish and German samples where the SSD and Raggedness Index or Raggedness Index was significant suggesting a stable or declining population.

### 3.5 Phylogeny

**Figure 5:**
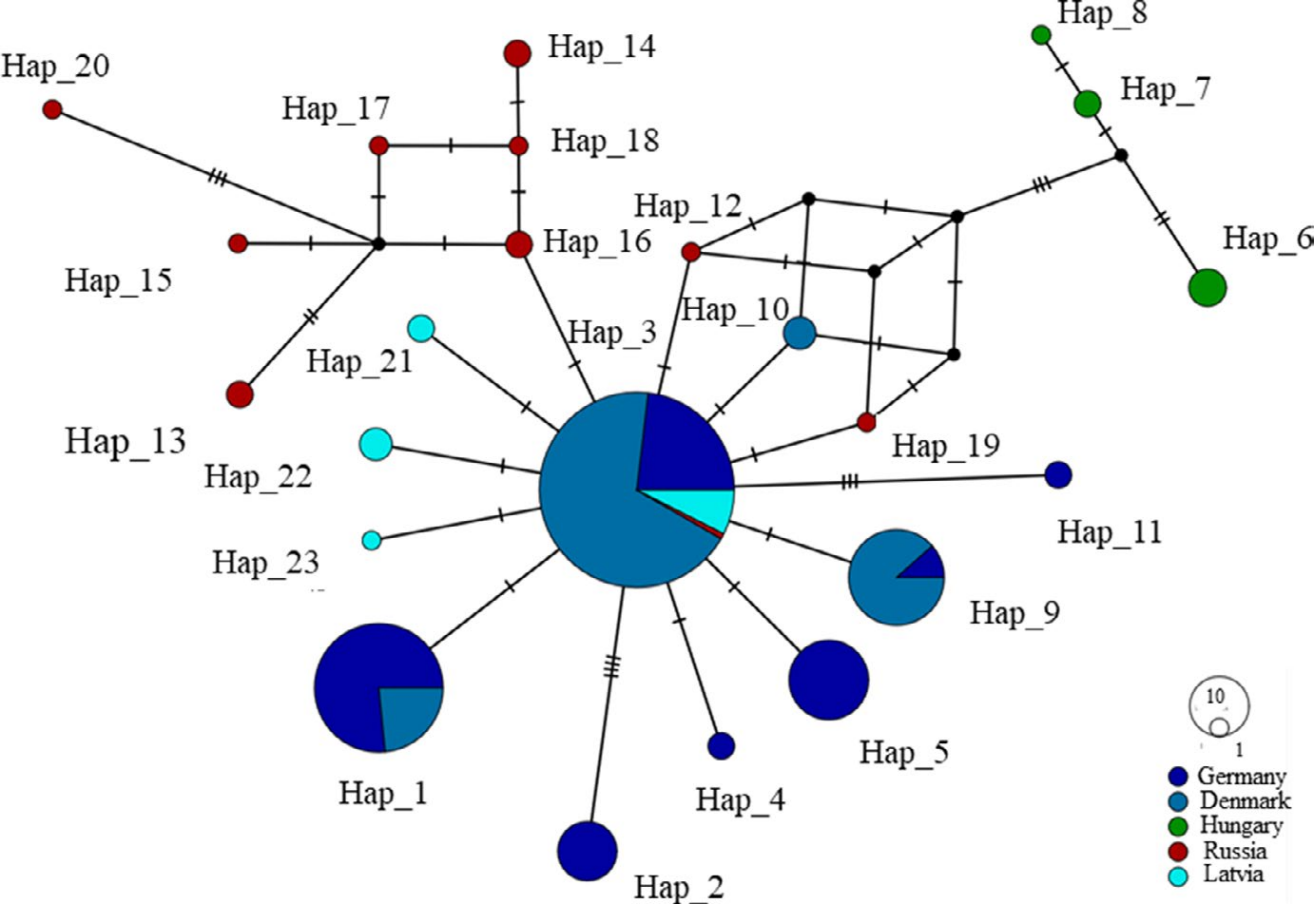
Median‐joining haplotype network of the concatenated CytB‐CR mtDNA sequences between pond bats from Denmark, Germany, Latvia, Hungary, and Russia indicating the phylogenetic relationships estimated using DnaSP (Librado & Rozas, 2009) and POPART (Leigh & Bryant, 2015). The size of the circles indicates the relative frequency of the haplotypes. The number of crossbars on the line connecting haplotypes indicates the number of mutation separating the haplotypes

The total number of observed concatenated haplotypes was 23 (CR 16 haplotypes, CytB 14 haplotypes; Supporting Information Appendix S5a,b). The relationship among the haplotypes reflected in the median‐joining network (Figure 5) revealed a close relationship between the Danish, German, and Latvian pond bats, while the Russian and Hungarian were more distantly related from all populations. The most common haplotype, Hap_3, was observed in all but the Hungarian sample. Denmark, Germany, and Latvia shared several haplotypes, and many of the other haplotypes represented in these samples were separated by just one mutation creating a starlike network characteristic for expanding populations that have been through a bottleneck or been founded recently.

**Figure 6:**
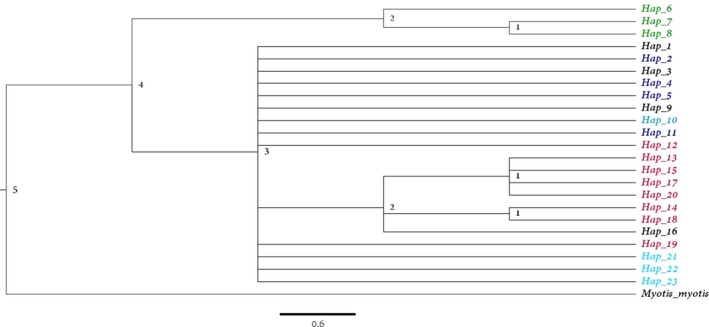
Phylogenetic consensus tree estimated in MrBayes 3.2.6 (Ronquist et al., 2012) indicating the genetic relationship between the haplotypes using the CytB and CR concatenated sequences from *Myotis myotis* as outgroup. Red = Russian haplotypes; black = haplotypes found in Denmark and Germany; blue = German haplotypes; light blue = Danish haplotypes; aqua azure = Latvian haplotypes; green = Hungarian haplotypes

The genetic relationship among the concatenated haplotypes analyzed using the Bayesian approach implemented in MrBayes 3.2.6 (Ronquist et al., 2012) (Figure 6) supported the distant relationship of the Hungarian and Russian pond bats compared to the Danish, German, and Latvian bats. The phylogeny showed four major clades: The first (5) separated *Myotis myotis* from the pond bat. The second (4) separated the Hungarian haplotypes, while the third (3) separated the Russian haplotypes from the other areas. Furthermore, a rather close relationship between the Danish and German pond bats was observed and the two populations shared some of the concatenated haplotypes.

## 4 DISCUSSION

This study provides new insights into the conservation genetics of the pond bat with emphasis on the genetic relationship between populations in northernmost Germany and in Jutland, Denmark.

Despite the lack of samples from some important pond bat populations in certain regions within the distribution range (i.e., the Netherlands, Belgium, and northernmost France), the present study offers a first indication of the genetic constitution of the species. A very close recent genetic relationship was revealed between the Danish and German populations by the microsatellite analysis. The multilocus pairwise *F*_ST_ and *D*_EST_ estimates were small but significant, and the Bayesian‐based cluster analysis did not identify Denmark and Germany as different clusters. This is probably a combined effect of the low number of microsatellite markers used but also due to migration connecting the two nearby populations. The concatenated mtDNA sequences suggested a clear genetic differentiation between all analyzed populations probably caused by genetic drift combined with founder effects as pond bats colonized Europe from different refugia after the last glacial period.

### 4.1 Genetic diversity

The level of genetic diversity observed at microsatellite loci in the Danish, German, and Russian pond bat populations was similar to or slightly lower than that found in the greater mouse‐eared bat (*Myotis myotis*) in the contact zone between European and Anatolian populations (average *H*_E_ = 0.74 and 0.61, respectively; Furman, Ҫelik, & Ҫoraman, 2018). The greater mouse‐eared bat is generally more sedentary and has typically shorter migration and dispersal distances compared to the pond bat (Corbet, 1978; Horáček, 1985). Ruedi and Castella (2003) studied 24 greater mouse‐eared bat colonies in southern Europe (3,000 km transect) using variation in CR sequences in the mitochondria. They observed 43 haplotypes in total (HD = 0.49, π ~ 0.03% − 2.08%, and 2–6 haplotypes in the different colonies), which was higher than the 23 different concatenated haplotypes (CytB‐CR) for pond bat in the present study. These diversity estimates were based on the control region exclusively; nevertheless, compared to the level for concatenated CytB‐CR sequences in the European pond bat groups, π observed in the latter was lower for all the analyzed groups while HD was in between (separated estimates for CR and CytB; Supporting Information Appendix S4). The rather high nucleotide diversity found in the Hungarian and Russian samples despite the low sampling size is probably due to sampling strategy, as pond bats were sampled over a wide range, representing several roosts within the countries. In contrast, the Latvian fecal samples were collected in the same roost and it is uncertain how many different individuals were sampled.

The low nucleotide diversity observed in the Danish group might reflect a recent colonization event compared to the other groups, as low genetic variation is expected in newly founded populations due to drift and reoccurring bottleneck/founding effects with limited gene flow during expansion of the species (Ramachandran et al., 2005). The limestone mines created by humans have provided suitable hibernation sites that would not have been available naturally in Denmark. The decreased genetic diversity with increasing distance from supposed Pleistocene refugia is not observed in the noctule bat, which probably can be attributed to the longer and regular migration distances of this species (Hutterer et al., 2005; Petit, Excoffier, & Mayer, 1999).

Pond bat bone remains have been recovered in Late Pleistocene caves in northern Italy outside the current range (Salari & Kotsakis, 2011), in Poland (summarized by Ciechanowski, Sachanowicz, & Kokurewicz, 2007), and near the easternmost recent range in the northwestern Altai in Central Asia (Rossina, 2006) (Figure 1). This suggests that the pond bat distribution probably was structured into several distinct populations during the last glacial period, as described for many other animal species (Taberlet, Fumagalli, Wust‐Saucy, & Cosson, 1998). Our genetic analyses corroborate these assumptions and the high nucleotide diversity indicates that the Hungarian population, despite the low sampling size, might be the most ancestral among the analyzed populations. To elaborate further on structuring and potential refugia for pond bats during the glacial periods, a more systematic widespread sampling covering the whole species' distribution is needed, including samples from the large westernmost pond bat population in the Netherlands and Belgium.

### 4.2 Population structure

The observed temporal effect in the Danish samples can most probably be ascribed to genetic drift caused by small sample sizes combined with the multigeneration time span separating the sampling episodes (estimated generation time = 5 years (Piraccini, 2016)). Despite this, the samples were aggregated analyzing the population structure to avoid noise related to the resulting unequal and small sample sizes from the temporal division. The Bayesian‐based population structure analysis in STRUCTURE detected two clusters, one including individuals from Denmark and Germany and another including Russian samples. However, it was difficult to identify those clusters from the graphical output. It is known that STRUCTURE has problems inferring the number of clusters when *F*_ST_ < 0.02 (Chen, Durand, Forbes, & Franòois, 2007; Latch, Dharmarajan, Glaubitz, & Rhodes, 2006) which is close to the levels of genetic differentiation observed between the areas. Pairwise *F*_ST_ as well as *D*_EST_ analysis did detect significant, however small, genetic differences between Denmark and Germany and Denmark and Russia, but not between Germany and Russia. However, the structuring pattern observed by the DAPC analysis segregated all three populations, suggesting a higher resolution using this method. The contradicting microsatellite results, found applying different methods, may indicate that the number of markers and sample sizes should be higher to resolve the structure properly.

The low, significant genetic differentiation reflected by the microsatellite markers between Danish and German pond bats compared to the Danish and Russian pond bat is probably caused by a higher male‐mediated gene flow due to geographical proximity.

Including Latvia and Hungary in the pairwise Φ_ST_ population structure analysis obtained from the concatenated mtDNA sequences revealed a pronounced structure supporting previous assumptions of female philopatric behavior in pond bat (Limpens et al., 2000). The observed differences in population structuring reflected by the two different marker types in the Danish and German pond bats are expected in a female philopatric species with intermediate dispersal behavior due to the different heritage pattern—the nuclear markers displaying the biparental contribution while the mitochondrial only signifies the female contribution. Furthermore, the more pronounced mtDNA differentiation can probably also be attributed to the maternal inheritance reducing effective populations size to one‐fourth that of nuclear genes, thus leading to the faster accumulation of allele frequency changes (DeSalle, Templeton, Mori, Pletscher, & Johnston, 1987). Last, the mtDNA data reflect the evolutionary history of the pond bat, that is, showing a stronger phylogeographical signal due to their relatively slower overall mutation rates (Hickerson et al., 2010).

The population structure pattern observed in the pond bat is concordant with population structure studies on Bechstein's bat (*Myotis bechsteinii*) and the noctule bat: the former showing strictly female philopatric behavior (Kerth, Mayer, & Petit, 2002) with male dispersal, the latter displaying less strictly female philopatry but seasonal long‐distance migration (Petit et al., 1999; Petit & Mayer, 1999,2000). In Bechstein's bat, *F*_STmic_ was not significant among the ten colonies analyzed while the Φ_STmt_ was highly significant (Kerth et al., 2002). In noctule bat, Petit et al. (Petit & Mayer, 1999,2000; 1999) observed a weak but significant *F*_STmic_, while Φ_STmt_ was higher and more pronounced between colonies compared to groups of colonies in Eastern and Central Europe (Petit et al., 1999; Petit & Mayer, 1999,2000).

The observed genetic population structure could be a reflection of “isolation by distance” (IBD), but no significant correlation was discovered, illustrating closer genetic relationship and shorter geographical distance between the populations. This may suggest that the genetic pattern indicates the existence of geographical barriers and/or historical colonization events.

### 4.3 Migration and detection of first‐generation migrants

The assignment test and detection of FGM between Danish and German populations illustrated a close genetic relationship between the two populations. The high percentage of inconclusive assignments to the populations can probably be attributed to the low number of markers used. However, some individuals sampled in the German roosts might be FGM from Denmark suggesting migration between the two areas, but a source–sink relationship was not detected. These results concur with the observations of the ringed German pond bat female that visited a hibernaculum in Denmark (Jagd & Artenschutz, 2010). Thus, dispersal between populations occupying hibernacula ca 300 kilometers apart do occur, supporting Ahlén et al.'s (2009) suggestion of pond bat behavior.

### 4.4 Population demography

Pond bat populations are assumed to be declining generally due to degradation and loss of feeding habitats and roosting sites (Piraccini, 2016). This might cause a significant reduction in population size and thus also in genetic diversity. However, this was not detected in the present study, which might be explained by the fact that the test used can only detect severe and recent bottlenecks (ca. 0.2–4.0 N_E_ generations; Luikart, 1998). In the Russian samples, the bottleneck test revealed a significant heterozygote excess, indicating a recent population bottleneck effect. This might reflect a response caused by reductions in population size as hypothesized, although the results should be interpreted with caution due to the low number of marker used and the low sample size. Chikhi, Sousa, Luisi, Goossens, and Beaumont (2010) showed that genetic differentiation/gene flow, genetic diversity, and the sampling scheme can generate false bottleneck signals.

Historically, the last glacial period ending ~11,600 years ago and the following recolonization influenced species distribution and genetic differentiation across Europe leaving genetic footprints in present species (Hewitt, 1999,2001). Ibanez, García‐Mudarra, Ruedi, Stadelmann, and Juste (2006) discovered deeply differentiated cryptic lineages by comparing mitochondrial sequences (cytochrome b and ND1) of Iberian and other European bat species, suggesting the Iberian Peninsula to be an important Ice Age refuge. For the greater mouse‐eared bat, Ruedi et al. (2008) showed that Italy was a major retreat area during glacial periods using variation in the control region of mtDNA. In the present study, different historical population demography signals were observed for the analyzed pond bat populations. The Russian sample showed a clear population expansion signal with a significant negative *F*_S_ and nonsignificant Raggedness Index. This supports the former suggested explanation that the bottleneck signal was false. In the Danish and German samples, SSDs were significant suggesting that the populations were either stable or declining (Rogers & Harpending, 1992). This population expansion pattern might reflect the historical colonization wave. In the most recent founded population, this signal will be replaced by a signal of stability or decline due to an increasing founder effect as observed in the Danish and German populations. This was supported by the observed pattern of genetic diversity—the older populations have higher genetic diversity (Handley, Manica, Goudet, & Balloux, 2007). Further, the phylogenetic inferences reflected by the median‐joining haplotype network showed a “starlike” haplotype network for the Danish and German samples, which is indicative for populations that have expanded from a bottleneck and small number of founders recently (Slatkin & Hudson, 1991).

The Bayesian phylogenetic consensus tree suggested a closer relationship between the Russian and Northern European pond bat populations compared to the Hungarian population. This might be indicative of Hungarian bats belonging to an older ancestral pond bat population; however, more samples should be included to verify this hypothesis.

### 4.5 Conservation implications

#### 4.5.1 Denmark and Germany

A significant genetic difference was observed between the two nearby populations in Denmark and Germany using both marker sets. However, the microsatellite analysis also reflected relatively high gene flow between Denmark and Germany. Further, assignment tests revealed the possibility that individuals caught in Germany could originate from the Danish population, as supported by the catching of a female pond bat in Germany that originally was ringed in Denmark. These results emphasize the need for cross‐border management of the species between these two countries to ensure future conservation.

#### 4.5.2 European level

From the mitochondrial analysis, our study documents clear genetic structuring of all the sampled pond bat populations, with unique haplotypes in most populations despite low sample sizes in some. Anthropogenic activities often cause species to decline, with habitat degradation and loss being the most important drivers at a large geographical scale (Frankham, Briscoe, & Ballou, 2010). For pond bats, the loss of roost sites and degradation of aquatic hunting habitats due to destruction and pollution are severe threats (Limpens et al., 2000). Changing climate puts further pressure on these populations as drier summers with less rainfall can alter the preferred hunting habitats and the occurrence of swarming insect populations (Meinig, 2010). Pond bats are rare and patchily distributed (Dietz et al., 2009; Krüger et al., 2014; Limpens et al., 2000), and the observations suggest a genetic structuring based on philopatric behavior with possible migration between nearby populations occurs. These results support the need to preserve and protect suitable habitat mosaics including underground sites to maintain a continuum of patches with dense pond bat populations to conserve genetic diversity in this species. This would further increase the probability of migration between populations across the whole of the species' distribution range. It is important to protect both hibernacula and the maternity roosts and the ecological functionality of the surrounding landscape. Matings during the swarming period in late summer at the large hibernacular sites may play a decisive role in ensuring gene flow between regional colonies in pond bats. Especially given the detected genetic relationship imply that females are philopatric to their maternity roosts (Bogdanowicz, Piksa, & Tereba, 2012; Furmankiewicz & Altringham, 2007; Kerth, Kiefer, Trappmann, & Weishaar, 2003; Kerth et al., 2002; Rivers, Butlin, & Altringham, 2005; Veith, Beer, Kiefer, Johannesen, & Seitz, 2004).

Further analyses of samples collected throughout the whole distribution range of pond bats (i.e., including samples from, e.g., Poland, Ukraine, Belgium, northern France, and the Netherlands) are needed to provide more information about the genetic structuring and colonization processes following the latest glacier period. Higher resolution genetic analysis, for example, involving RAD sequencing (Baird et al., 2008; Hohenlohe et al., 2010; Peterson, Weber, Kay, Fisher, & Hoekstra, 2012) would allow a better understanding of the migratory behavior and fine‐scale population structure of this species across regional and neighboring populations.

## ETHICAL STATEMENT

The Danish wing samples were collected in accordance with the Danish Animal Welfare Act. The German wing samples were collected by Dr Florian Gloza‐Rausch, scientific director of Noctalis, Bad Segeberg, Germany, under the permission and ethical approval provided by the State Agency for Agriculture, Environment and Rural Areas, Schleswig‐Holstein. The Hungarian wing samples were collected by Péter Estók under permission no. 14/2138‐7/2011.

## CONFLICT OF INTEREST

The authors declare no conflict of interests.

## AUTHORS' CONTRIBUTIONS

LWA, RD, HJB, FGR, FK, and ME conceived the study. RD, HJB, GP, PE, OLO, MO, MG, FK, and ME did the fieldwork. LWA, RD, EN, and MO did laboratory analysis. LWA and RD conducted the data analysis with contribution from EN. FGR secured funding. All authors helped drafting the manuscript and gave approval before publication.

## Data Availability

All sequences are deposited in GenBank with accession numbers (CR) MK598578–MK598593 and (CytB) MK603162–MK603174. Hap_1–Hap_23 are concatenated from CR and CytB with the following combination of accession numbers from Hap_1: MK598578, MK603170; MK598581, AF376846.1; MK598580, MK603170; MK598580, MK603173; MK598582, MK603170; MK598584, MK603163; MK598585, MK603162; MK598586, MK603162; MK598580, MK603171; MK598583, MK603170; MK598579, MK603170; MK598589, MK603170; MK598590, MK603165; MK598591, MK603174; MK598592, MK603164; MK598580, MK603168; MK598591, MK603164; MK598591, MK603168; MK598588, MK603170; MK598593, MK603166; MK598580, MK603167; MK598580, MK603172; Hap_23: MK598580, MK603169. The microsatellite genotypes data are deposited at Dryad Digital Repository https://doi.org/10.5061/dryad.cq11s60.
