# Supplementary material for: Conservation genetics of the pond bat (Myotis dasycneme) with special focus on the populations in northwestern Germany and in Jutland, Denmark
Source: Ecol Evol. 2019 Apr 1;9(9):5292–308. doi: 10.1002/ece3.5119 (PMC6509384; doi:10.1002/ece3.5119)
Supplement: Supplementary file 1 [file ECE3-9-5292-s001.docx]

**Conservation genetics of the pond bat (Myotis dasycneme) with special focus on the populations in northwestern Germany and Jutland, Denmark**

Andersen Liselotte Wesley ^1^, Dirksen Ronja^2^, Nikulina Elena A^3^, Baagøe Hans J.^4^, Petersons Gunars^5^, Estók Péter^6^, Orlov Oleg L^7^, Orlova Maria V^8^, Gloza-Rausch Florian^9^ , Göttsche Matthias^10^ , Fjederholt Esben Terp^11^, Krüger Frauke^12^, Elmeros Morten^1^

Appendix 1. Pond bat assessment in Latvia, Hungary an Russia

In Latvia, the pond bat is relatively rare, most abundant in the south-eastern part of the country. About 20 nursery colonies are known, majority of them roost in churches. The total number of adult females in colonies is more than 1500 individuals according counts during emergence. At hibernation sites the pond bat is rare, and only in two, their number exceeds 50 individuals (Petersons & Vintulis, 1998). The conservation status of the species in Latvia is assessed as moderately unfavourable ([www.eionet.eu](http://www.eionet.eu)). In Hungary, the species’ main distribution area is near large rivers and lakes in lowlands. Nurseries can be found in lowland forests, in attics and in bridges, with the biggest colony consisting of 2000 individuals. During swarming, pond bats visit caves in mountainous regions. Limited data of hibernating individuals are known from caves, it is hypothesised, that part of the population hibernates in tree hollows (Görföl & Estók, 2014). The population is declining and its conservation status is moderately unfavourable due to inadequate habitats ([www.eionet.eu](http://www.eionet.eu); [www.eurobats.org](http://www.eurobats.org)). The pond bat population seems to be stable in Russia, but there is currently little information on the population status in the eastern parts of the species range in Russia ([www.eurobats.org](http://www.eurobats.org); Piraccini, 2016).

Appendix 2. Microsatellite primers, dye label and optimal annealing temperature. TA : optimal annealing temperature use to analyse population structure of pond bats (*Myotis dasycneme*) in Europe.

| Primer | Sequence 5’-3’ |  | Label | TA [°C] |
| --- | --- | --- | --- | --- |
| A13F | AACGTTCATTCTGCCAAAGG | | NED | 56 |
| A13R | TCATGCTGTTCCACTTCTGG | |  |  |
| D9F | TCTTTCCTCCCCTGTGCT | | FAM | 65 |
| D9R | TCTGGACCCAAAATGCAGG | |  |  |
| D15F | GCTCTCTGAAGAGGCCCT | | NED | 56 |
| D15R | ATTCCAAGAGTGACAGCATCC | |  |  |
| E24F | GCAGGTTCAATCCCTGACC | | FAM | 56 |
| E24R | AAAGCCAGACTCCAAATTCTG | |  |  |
| F19F | GCTAGCCATGGAGAAGGAAG | | FAM | 56 |
| F19R | CCCAAATCTGTCTTTCAGGC | |  |  |
| G9F | AGGGGACATACAAGAATCAACC | | FAM | 56 |
| G9R | TAATTTCTCCACTGAACTCCCC | |  |  |
| G25F | TCCTTCCCATTTCTGTGAGG | | NED | 56 |
| G25R | CCATTTCATCCATCCAGTCC | |  |  |
| G30F | TTGCCAAATTCTGGTATCTTCC | | VIC | 60 |
| G30R | AGAGCTTAATGGGGAGGCTG | |  |  |
| H19F | GGAATCCGAATCCCTGGC | | FAM | 63 |
| H19R | GACATCCCCTCACCCCAAC | |  |  |
| H29F | TCAGGTGAGGATTGAAAACAC | | VIC | 56 |
| H29R | GCTTTATTTAGCATTGGAGAGC | |  |  |
| All primers from Castella & Ruedi (2000) | | |  |  |

Appendix 3. The 10 microsatellite markers used to study pond bat in Europa. N= sample size, N_a_= number of different alleles, H_o_ observed heterozygosity, H_e_ expected heterozygosity (GenAlEx, Peakall & Smouse 2006, 2012), F_is_ deviations from HWE (FSTAT, Goudet, 1995).

|  |  | *A13* | *E24* | *G9* | *f19* | *d9* | *G25* | *H19* | *H29* | *d15* | *g30* | Mean | SE |
| --- | --- | --- | --- | --- | --- | --- | --- | --- | --- | --- | --- | --- | --- |
| Denmark |  |  |  |  |  |  |  |  |  |  |  |  |  |
| Mønsted 2003 | N | 51 | 51 | 51 | 51 | 50 | 51 | 51 | 51 | 51 | 51 |  |  |
|  | N_a_ | 10 | 9 | 8 | 13 | 9 | 1 | 2 | 9 | 8 | 4 |  |  |
|  | H_o_ | 0.882 | 0.902 | 0.745 | 0.941 | 0.740 | 0.000 | 0.451 | 0.784 | 0.706 | 0.314 | 0.647 | 0.095 |
|  | H_e_ | 0.796 | 0.841 | 0.791 | 0.886 | 0.828 | 0.000 | 0.462 | 0.834 | 0.796 | 0.284 | 0.652 | 0.095 |
|  | F_is_ | -0.099 | -0.063 | 0.068 | -0.052 | 0.116 | NA | 0.034 | 0.069 | 0.123 | -0.096 | 0.018 |  |
| Mønsted 2011 | N | 18 | 16 | 19 | 19 | 19 | 19 | 19 | 18 | 19 | 19 |  |  |
|  | N_a_ | 10 | 10 | 8 | 10 | 9 | 1 | 2 | 11 | 6 | 5 |  |  |
|  | H_o_ | 0.833 | 0.563 | 0.737 | 0.842 | 0.684 | 0.000 | 0.368 | 0.944 | 0.421 | 0.684 | 0.608 | 0.089 |
|  | H_e_ | 0.799 | 0.818 | 0.738 | 0.871 | 0.810 | 0.000 | 0.478 | 0.850 | 0.640 | 0.586 | 0.659 | 0.084 |
|  | F_is_ | -0.014 | 0.341 | 0.029 | 0.06 | 0.182 | NA | 0.254 | -0.082 | 0.366 | -0.141 | 0.106 |  |
| Daugbjerg 2009 | N | 38 | 38 | 38 | 38 | 38 | 38 | 38 | 38 | 36 | 34 |  |  |
|  | N_a_ | 10 | 14 | 11 | 12 | 12 | 1 | 2 | 10 | 7 | 6 |  |  |
|  | H_o_ | 0.684 | 0.868 | 0.921 | 0.816 | 0.789 | 0.000 | 0.395 | 0.868 | 0.750 | 0.382 | 0.647 | 0.093 |
|  | H_e_ | 0.804 | 0.861 | 0.808 | 0.883 | 0.760 | 0.000 | 0.458 | 0.857 | 0.779 | 0.436 | 0.665 | 0.090 |
|  | F_is_ | 0.162 | 0.005 | -0.127 | 0.089 | -0.025 | NA | 0.151 | 0 | 0.051 | 0.138 | 0.039 |  |
| Daugbjerg 2011 | N | 11 | 12 | 12 | 12 | 12 | 12 | 12 | 12 | 12 | 12 |  |  |
|  | N_a_ | 8 | 7 | 7 | 8 | 6 | 1 | 2 | 7 | 5 | 5 |  |  |
|  | H_o_ | 0.727 | 0.833 | 1.000 | 1.000 | 0.500 | 0.000 | 0.167 | 0.833 | 0.500 | 0.417 | 0.598 | 0.108 |
|  | H_e_ | 0.773 | 0.792 | 0.816 | 0.840 | 0.799 | 0.000 | 0.486 | 0.840 | 0.611 | 0.476 | 0.643 | 0.084 |
|  | F_is_ | 0.106 | -0.009 | -0.184 | -0.148 | *0.411* | NA | 0.681 | 0.052 | 0.224 | 0.167 | 0.114 |  |
| Denmark total | N | 118 | 117 | 120 | 120 | 119 | 120 | 120 | 119 | 118 | 116 |  |  |
|  | N_a_ | 12 | 15 | 12 | 13 | 13 | 1 | 2 | 11 | 9 | 8 |  |  |
|  | H_o_ | 0.797 | 0.838 | 0.825 | 0.892 | 0.723 | 0 | 0.392 | 0.84 | 0.653 | 0.405 | 0.636 | 0.09 |
|  | H_e_ | 0.837 | 0.847 | 0.805 | 0.902 | 0.815 | 0 | 0.467 | 0.852 | 0.825 | 0.409 | 0.676 | 0.092 |
|  | F_is_ | 0.052 | 0.015 | -0.02 | 0.015 | ***0.117*** | NA | 0.165 | 0.018 | ***0.213*** | 0.013 | 0.063 |  |
|  |  | *A13* | *E24* | *G9* | *f19* | *d9* | *G25* | *H19* | *H29* | *d15* | *g30* | Mean | SE |
| Germany | N | 81 | 80 | 81 | 81 | 81 | 81 | 81 | 81 | 81 | 81 |  |  |
|  | N_a_ | 10 | 9 | 12 | 11 | 12 | 2 | 2 | 12 | 8 | 8 |  |  |
|  | H_o_ | 0.864 | 0.7 | 0.815 | 0.827 | 0.852 | 0.012 | 0.457 | 0.926 | 0.79 | 0.272 | 0.651 | 0.096 |
|  | H_e_ | 0.821 | 0.829 | 0.821 | 0.88 | 0.873 | 0.012 | 0.463 | 0.87 | 0.817 | 0.347 | 0.673 | 0.094 |
|  | F_is_ | -0.047 | 0.162 | 0.013 | 0.066 | 0.03 | 0 | 0.02 | -0.058 | 0.039 | 0.222 | 0.038 |  |
| Russia | N | 23 | 23 | 23 | 23 | 23 | 23 | 23 | 23 | 22 | 22 |  |  |
|  | N_a_ | 8 | 9 | 14 | 11 | 8 | 1 | 2 | 10 | 9 | 2 |  |  |
|  | H_o_ | 0.696 | 0.913 | 0.826 | 0.913 | 0.783 | 0 | 0.565 | 0.87 | 0.682 | 0.227 | 0.647 | 0.097 |
|  | H_e_ | 0.812 | 0.81 | 0.863 | 0.866 | 0.804 | 0 | 0.44 | 0.841 | 0.834 | 0.325 | 0.659 | 0.095 |
|  | F_is_ | 0.165 | -0.105 | 0.065 | -0.032 | 0.049 | NA | -0.265 | -0.011 | 0.205 | 0.323 | 0.041 |  |

Global allele number 13 15 16 13 14 2 2 13 11 11

|  |  |  |  |  |  |  |  |  |  |  |  |
| --- | --- | --- | --- | --- | --- | --- | --- | --- | --- | --- | --- |

Appendix 4. Sample size (N) and genetic diversity is given as number of mtDNA haplotypes (H), haplotype diversity (HD), nucleotide diversity ($\pi$; Nei, 1987) for control region (CR) and CytB separated. Tests for selective neutrality, Tajima’s D (Tajima, 1989) and Fu’s *F_s_* (Fu, 1997)(in ARLEQUIN Excoffier & Lischer, 2010) for the five different pond bat regions were performed and further population expansion indices based on CR and CytB sequences in terms of SSD= sum of squares deviations between observed and expected mismatch and Ragg. Id. = Raggedness Index of the mismatch distribution were analysed (ARLEQUIN, Excoffier & Lischer, 2010). **Bold**= significant at the 5% level.

|  |  | MON03 | MON11 | DAU09 | DAU11 | Denmark Total | Germany | Latvia | Hungary | Russia |
| --- | --- | --- | --- | --- | --- | --- | --- | --- | --- | --- |
| Control Region (CR) | N | 48 | 20 | 34 | 12 | 114 | 103 | 25 | 8 | 24 |
|  | H | 3 | 1 | 3 | 2 | 3 | 5 | 1 | 3 | 8 |
|  | HD | 0.228 | NA | 0.314 | 0.303 | 0.222 | 0.719 | 0 | 0.714 | 0.841 |
|  | SE | 0.075 | NA | 0.095 | 0.147 | 0.004 | 0.002 | 0 | 0.04 | 0.01 |
|  | π (%) | 0.09 | NA | 0.13 | 0.12 | 0.09 | 0.53 | 0 | 0.41 | 0.57 |
|  | TajimaD | -0.8733 | NA | -0.6506 | -0.1949 | -0.594 | -0.083 | 0 | 1.103 | -0.788 |
|  | Fu's *Fs* | -1.1178 | NA | -0.6642 | 0.29736 | -0.778 | 1.351 | 0 | 0.204 | **-3.07** |
| Spatial expansion | SSD | **0.0009** | **NA** | **0.0027** | **0.0019** | **0.0008** | 0.002 | 0 | 0.015 | **0.017** |
|  | Rag | 0.3491 | NA | 0.2312 | 0.247 | 0.359 | 0.044 | 0 | 0.122 | **0.118** |
| Demographic expansion did not converge | | |  |  |  |  |  |  |  |  |
|  |  |  |  |  |  |  |  |  |  |  |
|  |  | MON03 | MON11 | DAU09 | DAU11 | Denmark Total | Germany | Latvia | Hungary | Russia |
| CytB | N | 50 | 18 | 34 | 12 | 114 | 106 | 19 | 7 | 14 |
|  | H | 2 | 2 | 2 | 2 | 2 | 4 | 4 | 2 | 6 |
|  | HD | 0.35 | 0.425 | 0.299 | 0.303 | 0.335 | 0.255 | 0.614 | 0.517 | 0.868 |
|  | SE | 0.066 | 0.099 | 0.085 | 0.147 | 0.004 | 0.005 | 0.022 | 0.045 | 0.014 |
|  | π (%) | 0.07 | 0.08 | 0.06 | 0.06 | 0.06 | 0.08 | 0.14 | 0.22 | 0.29 |
|  | TajimaD | 0.764 | 0.87 | 0.3363 | -0.1949 | 0.883 | -0.781 | -0.459 | 1.649 | -0.099 |
|  | Fu's *Fs* | 1.2256 | 1.0391 | 0.7854 | 0.2974 | 1.509 | -0.538 | -0.823 | 2.047 | -1.704 |
| Spatial expansion | SSD | **0.0038** | **0.0098** | **0.0018** | **0.0019** | **0.003** | 0.012 | **0.034** | **0.178** | 0.011 |
|  | Rag | 0.2124 | 0.2031 | 0.2505 | 0.247 | 0.220 | 0.469 | 0.199 | 0.837 | 0.115 |
| Demographic expansion did not converge | | |  |  |  |  |  |  |  |  |

Appendix 5. Median-joining haplotype network of the a) control region and b) CytB sequences between pond bats from Denmark, Germany, Latvia, Hungary and Russia indicating the phylogenetic relationships estimated using DnaSP (Librado & Rozas, 2009) and POPART (Leigh & Bryant, 2015)(Pb_Gec2= GenBank accession no AF376846.1, Ruedi & Mayer (2001)). The size of the circles indicates the relative frequency of the haplotypes. The number of cross bars on the line connecting haplotypes indicates the number of mutation separating the haplotypes.

a)


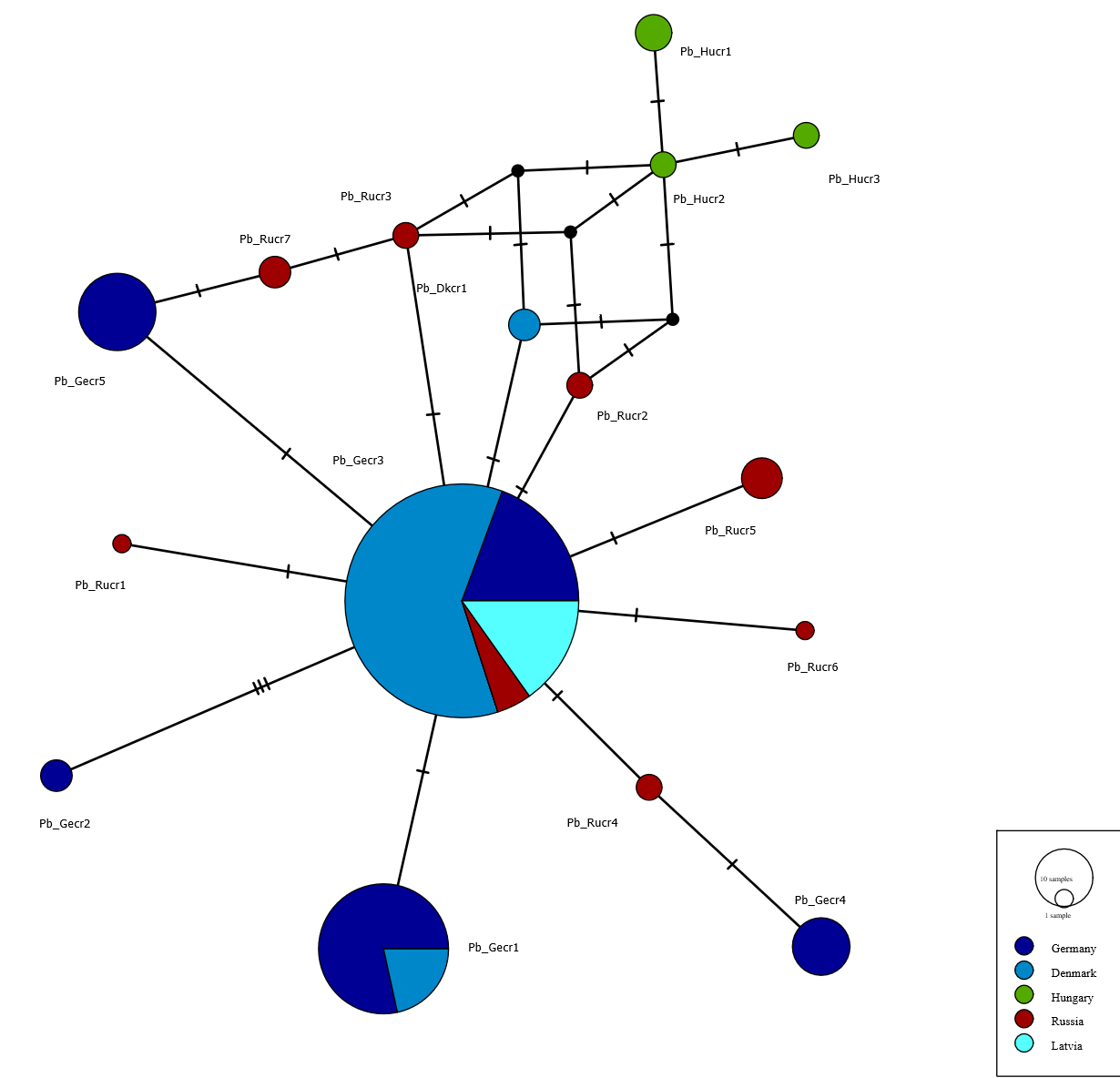


b)


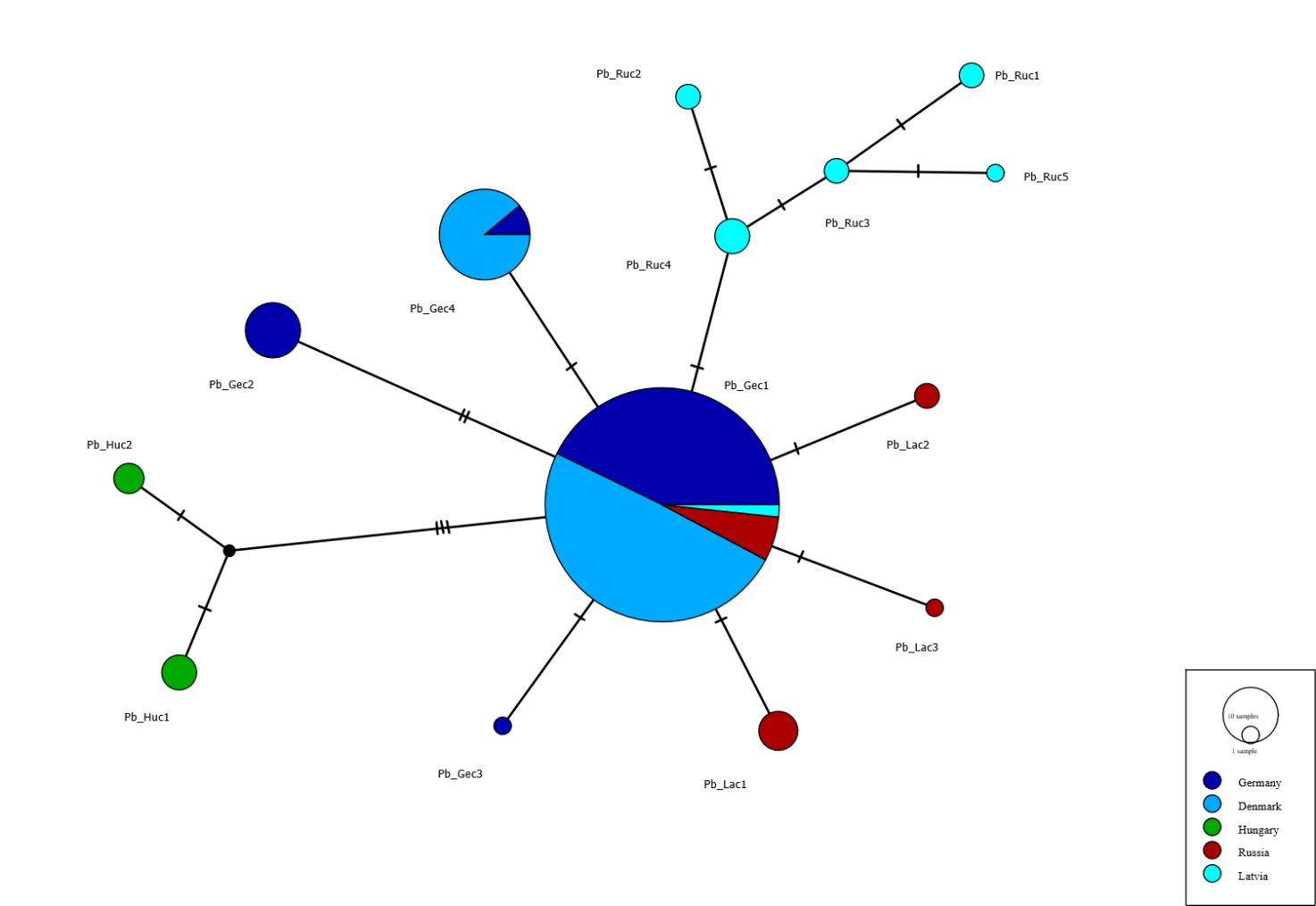


Appendix 6. Genetic divergence estimated between the five geographical areas. a) Pairwise Φ_ST_ results (pairwise distance) based on CR and b) CytB sequences from the five geographically regions with pond bat data. Bold values are significant after sequential Bonferroni correction (Rice, 1989).

| b) Control region (CR) | |  |  |  |  |
| --- | --- | --- | --- | --- | --- |
|  | Denmark | Germany | Latvia | Hungary |  |
| Germany | **0.151** |  |  |  |  |
| Latvia | 0.028 | **0.145** |  |  |  |
| Hungary | **0.923** | **0.726** | **0.936** |  |  |
| Russia | **0.218** | **0.148** | **0.118** | **0.677** |  |
| c) CytB |  |  |  |  |  |
|  | Denmark | Germany | Latvia | Hungary |  |
| Germany | **0.115** |  |  |  |  |
| Latvia | **0.23** | **0.15** |  |  |  |
| Hungary | **0.903** | **0.87** | **0.811** |  |  |
| Russia | **0.639** | **0.563** | **0.434** | **0.747** |  |

REFERENCES

Castella, V., Ruedi, M. (2000). Characterization of highly variable microsatellite loci in the bat *Myotis myotis* (Chiroptera: Vespertilionidae). *Molecular Ecology* 7, 1000–1002.

Excoffier, L., & Lischer, H.E.L. (2010). Arlequin suite ver. 3.5: a new series of programs to perform population genetics analyses under Linux and Windows. *Molecular Ecology Resources* 10, 564–567.

Fu, Y.X. (1997). Statistical neutrality of mutations against population growth, hitchhiking and background selection. *Genetics* 147,915-925.

Goudet, J. (1995). FSTAT 2.9.3.1: a computer program to calculate F statistics. *Journal of Heredity*

86, 485–486.

Görföl, T., & Estók, P. (2014). Tavi denevér – Myotis dasycneme (Boie, 1825). – In: Haraszthy L. (ed.): *Natura 2000 fajok és élőhelyek Magyarországon. Pro Vértes Természetvédelmi Közalapítvány, Csákvár*, 669–671.

Librado, P., & Rozas, J. (2009). DnaSP v5: a software for comprehensive analysis of DNA polymorphism data. *Bioinformatics* 25, 1451–1452

Peakall, R., & Smouse, P.E. (2006). GENALEX 6: genetic analysis in Excel. Population genetic software for teaching and research. *Molecular Ecology Notes* 6, 288-295.

Peakall, R., & Smouse, P.E. (2012). GenAlEx 6.5: genetic analysis in Excel. Population genetic software for teaching and research-an update. *Bioinformatics* 28, 2537-2539.

Pētersons, G., & Vintulis, V. (1998). Distribution and status of bats in Latvia*. Proceedings of the Latvian Academy of Sciences,* Section B, 52, 37-43.

Piraccini, R. (2016). *Myotis dasycneme*. The IUCN Red List of Threatened Species, e.T14127A22055164. <http://dx.doi.org/10.2305/IUCN.UK.2016-2.RLTS.T14127A22055164.en>

Ruedi, M. & Mayer,F. (2001). Molecular systematics of bats of the genus Myotis (Vespertilionidae) suggests deterministic ecomorphological convergences. *Molecular Phylogenetics and Evolution* 21, 436-448.

Tajima, F. (1989). Statistical methods to test for nucleotide mutation hypothesis by DNA polymorphism. *Genetics* 123, 585-595.
